# Feasibility, Stability, and Clinical Outcomes of Mucosal Defect Closure Using a Novel Hooking-Jaw Clip after Gastric Endoscopic Submucosal Dissection

**DOI:** 10.1055/a-2925-5683

**Published:** 2026-08-13

**Authors:** Takeshi Shimizu, Taku Yamagata, Yoshihide Kanno, Tomohiro Shimada, Kai Korekawa, Hiroki Sato, Yutaka Eto, Yosei Sawai, Teruyuki Numata, Kei Ito

**Affiliations:** 1 Department of Gastroenterology 506803 Public Interest Incorporated Foundation Sendai City Medical CenterSendaiMiyagi PrefectureJapan

**Keywords:** endoscopy upper GI tract, endoscopic resection (ESD, EMRc, ...), precancerous conditions and cancerous lesions (dysplasia and cancer) stomach, ulcers (peptic and other)

## Abstract

**Background and Study Aims** Clipping is a simple conventional closure technique for gastric endoscopic submucosal dissection (ESD) defects. This study evaluated the technical feasibility and the stability of mucosal defect closure using a novel hooking-jaw clip. In addition, we assessed the safety outcomes compared with a nonclosure group.

**Patients and Methods** This single-center retrospective pilot study assessed technical success (complete closure, ≥90% area reduction) and closure stability during second-look endoscopy (SLE) on postoperative day 1 in 41 patients. We compared the secondary outcomes with a nonclosure group using propensity score matching (PSM) ( *n* = 41 per group). Secondary outcomes included incidences of delayed bleeding and frequency of hemostatic interventions during SLE.

**Results** Hooking-jaw clip-closure achieved technical success in 92.7% (38/41) of patients; partial closure (50%–89% area reduction) occurred in 7.3% (3/41), and no closure failure (<50%) occurred. The median closure time was 10.0 min. Closure remained stable in 87.8% (36/41) of patients at SLE. After PSM, the delayed bleeding rate was 0.0% (0/41) in the closure group and 4.9% (2/41) in the nonclosure group ( *P* = 0.494). The closure group required a lower rate of hemostatic interventions during SLE (24.4% [10/41] vs. 58.5% [24/41]; *P* = 0.003).

**Conclusions** Hooking-jaw clip-assisted closure of gastric ESD defects is technically feasible and maintains stability. Closing reduced the frequency of hemostatic interventions during SLE. Given current guidelines that conditionally recommend omitting routine SLE, stable closure provides a physical rationale to support this practice. Future trials should evaluate this strategy for high-risk populations.

## Introduction

Delayed bleeding is a significant adverse event following gastric endoscopic submucosal dissection (ESD). Although ESD has become a widely practiced treatment for early gastric cancer, postoperative bleeding remains challenging. Several factors, such as large mucosal defects and the use of antithrombotic agents, increase this risk. 1

Closing the mucosal defect is a potential strategy to reduce the incidence of delayed bleeding. 2 Various closure techniques have been reported, including simple clipping and suturing with dedicated devices. 3 4 5 6 7 8 However, these methods often involve complex preparations or are expensive, limiting their widespread use in daily practice.

**Fig. 1:**
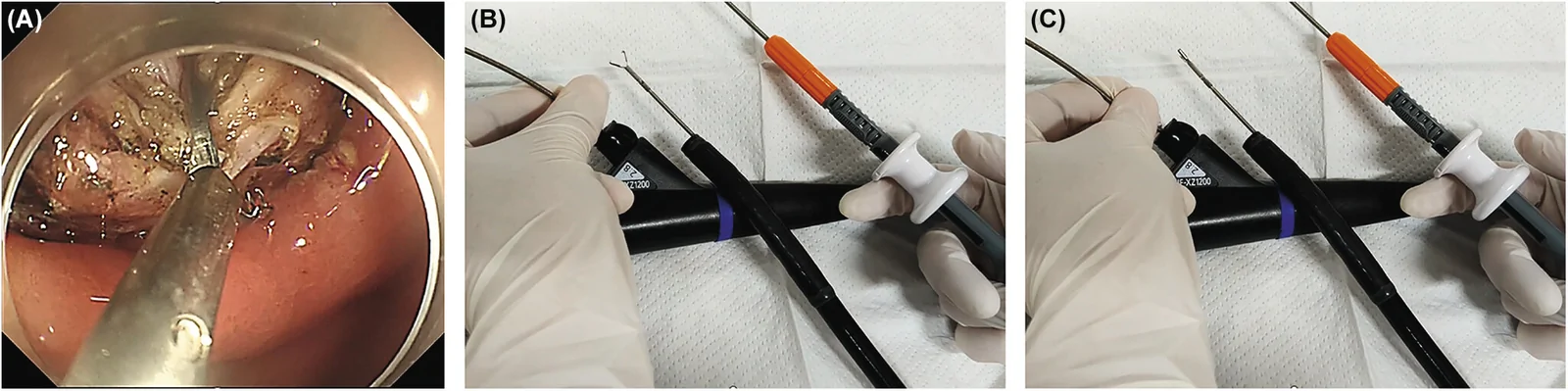
Device features of the hooking-jaw clip (MANTIS clip). ( **A** ) The MANTIS clip features a unique bifurcated hooking jaw securely grasping the tissue, enabling firm traction over long distances for effective mucosal defect closure after gastric endoscopic submucosal dissection. ( **B** , **C** ) Its rotatable head that allows endoscopists to freely manipulate and rotate the clip near the forceps channel.

A new clipping device, MANTIS clip (Boston Scientific, Marlborough, USA), offers potential advantages for this procedure. The MANTIS clip features a strong gripping power and a unique hooking-jaw design for securely hooking tissues ( Fig. 1A ). In addition, it features a rotatable design, allowing precise positioning by both assistants and endoscopists ( Fig. 1B,C ). Despite these features, no comparative studies have evaluated the clinical outcomes of defect closure in gastric ESD using a MANTIS clip.

Effective closure may reduce the need for second-look endoscopy (SLE). SLE is commonly performed the day after ESD to prevent bleeding; however, recent studies suggest that it may not reduce delayed bleeding. 9 10 11 Reflecting this evidence, current guidelines conditionally recommend the omission of routine SLE after gastric ESD. 12 Although the clinical value of routine SLE is generally considered limited, its utility specifically in high-risk patients remains a subject of controversy. Furthermore, emerging evidence indicates that routine SLE may serve as a mechanical trigger for iatrogenic hemorrhage due to procedural irritation of the ulcer bed. 9 10 If MANTIS clip-assisted closure can effectively approximate high-risk defects into a stable state, it may provide a physical shield against such mechanical irritation, potentially offering a rationale to support the omission of SLE.

This pilot study evaluated the technical feasibility and stability of MANTIS clip-assisted closure. We assessed the technical success and stability of the closure, as well as clinical outcomes, such as the frequency of hemostatic interventions during SLE and delayed bleeding, by comparing cases treated with MANTIS clip closure to those in a nonclosure group.

## Patients/Material and Method

### Study Design

This was a single-center, nonrandomized, retrospective pilot study conducted at Sendai City Medical Center. The study was approved by the Institutional Review Board (Approval Number: 2024-0080). The requirement for written informed consent was waived by the Institutional Review Board due to the retrospective design of the study, and an opt-out approach was utilized instead.

### Patients

Patients aged ≥18 years with histologically confirmed gastric adenocarcinoma or adenoma who underwent gastric ESD at our institution between January 2023 and February 2024 were included in this study. SLE was generally performed the day following ESD. We excluded patients who met any of the following criteria: (1) simultaneous resection of two or more lesions, (2) defect closure performed without using the MANTIS clip, and (3) failure to perform SLE within 3 days after ESD.

Eligible patients were categorized into two groups based on the management of the mucosal defect. The closure group included patients in whom defect closure was attempted using the MANTIS clip. The nonclosure group consisted of patients in whom the mucosal defects were left open, including cases in which conventional clips were used solely for hemostasis or perforation repair. All patients received vonoprazan 20 mg orally as standard post-ESD acid suppression therapy.

### ESD Procedure

ESD was performed by experienced endoscopists and trainees under the supervision of experienced endoscopists, using standard techniques. The procedures were conducted using high-definition endoscopes and video systems, including GIF-H290T, GIF-Q260TM, and EVIS X1 (Olympus, Tokyo, Japan), as well as EG-840TP, EG-840T, and ELUXEO 7000 (Fujifilm, Tokyo, Japan). For submucosal dissection, the choice of endoknife was determined by the endoscopist, and ORISE ProKnife (Boston Scientific), DualKnife-J (Olympus), and IT-Knife 2 (Olympus) were used as necessary. Submucosal injection was performed using a 0.4% hyaluronic acid solution, diluted with normal saline at the discretion of the endoscopist. Sedation and analgesia were administered according to standard protocols. Primary sedation was achieved through continuous intravenous infusion of propofol, whereas pentazocine was used for analgesia. If additional sedation was required, midazolam and/or droperidol were administered. Resection time was defined as the duration from the start of submucosal injection to the completion of lesion removal.

### Closure Procedure



**Video 1** Representative cases of post-endoscopic submucosal dissection (ESD) mucosal defect closure using the hooking-jaw clip (MANTIS clip). **Case 1: Complete closure.** Post-ESD mucosal defect before closure. Conventional clips are applied diagonally along the defect margins to reshape the defect into an oval. A MANTIS clip is placed centrally with deep grasping of the muscular layer to eliminate submucosal dead space. Additional conventional clips are applied to the remaining gaps, achieving complete closure (≥90% area reduction). Second-look endoscopy (SLE) on post-ESD day 1 demonstrates sustained closure without dehiscence. **Case 2: Partial closure.** A large post-ESD mucosal defect spanning the angulus along the lesser curvature. Conventional clips are applied diagonally to reshape the defect. A MANTIS clip is placed centrally with firm grasping of the muscular layer. Partial closure (50–89% area reduction) is achieved. SLE on post-ESD day 1 demonstrates stable wound condition without dehiscence.


Closure was performed after completion of lesion resection and hemostatic coagulation for vascular control. The indication for closure was defined as a mucosal defect with a major axis visually estimated to be less than 60 mm, and closure was performed at the discretion of the attending expert endoscopist based on operator availability and scheduling at the time of the procedure. Closure was not performed consecutively but depended on the availability of expert endoscopists. All closure procedures in the closure group were performed by expert endoscopists (>300 gastric ESD experience).

**Fig. 2:**
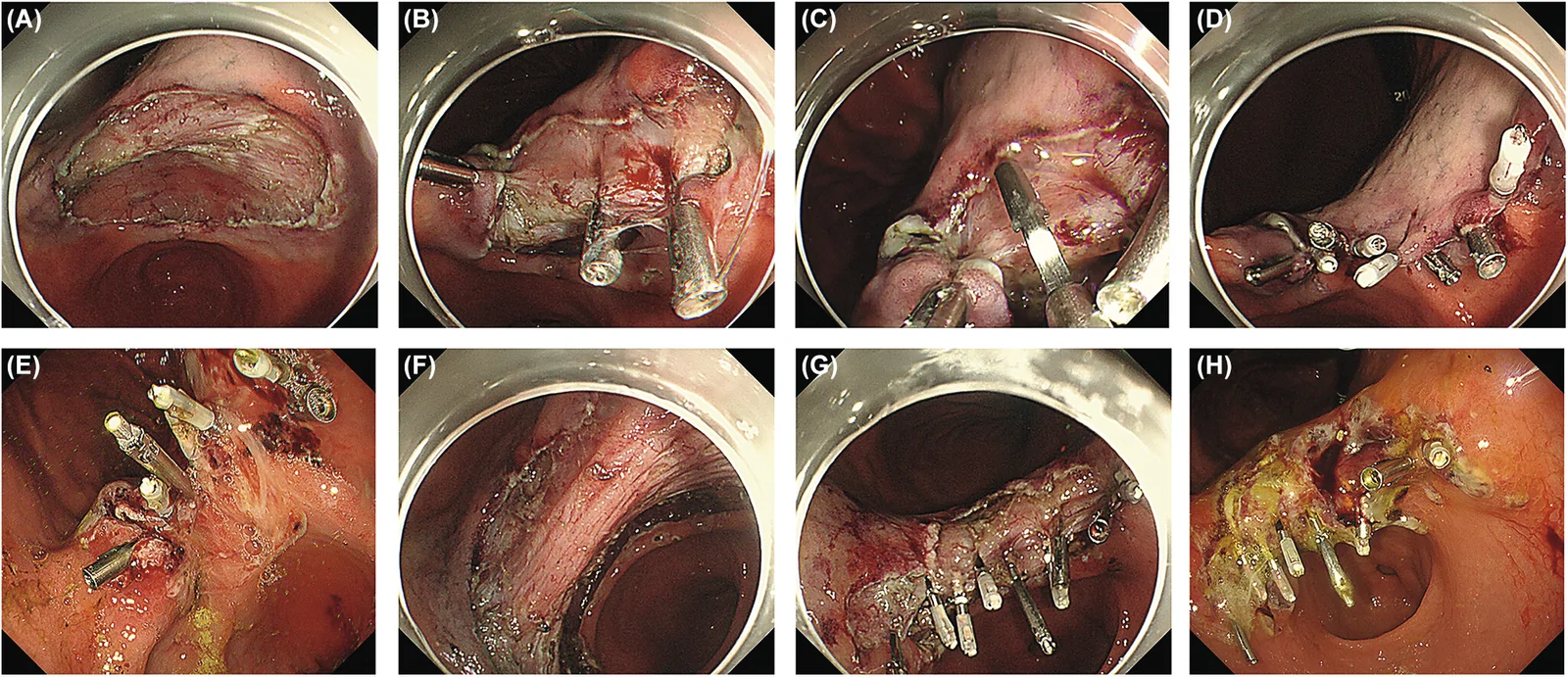
Representative cases of mucosal defect closure using the hooking-jaw clip. ( **A** – **E** ) Complete closure case. ( **A** ) Post-endoscopic submucosal dissection (ESD) mucosal defect before closure. ( **B** ) Conventional clips are applied diagonally along the margins of the defect, converting it from a circular to an oval shape. ( **C** ) A MANTIS clip is placed at the center of the narrowed defect, with deep grasping of the opposing muscular layers to eliminate submucosal dead space and ensure firm wound apposition. ( **D** ) Additional conventional clips are placed to achieve complete closure (≥90% of the defect is closed) of the remaining gaps. ( **E** ) Second-look endoscopy (SLE) on post-ESD day 1 demonstrating sustained closure with no dehiscence. ( **F** – **H** ) Partial closure case. ( **F** ) A large post-ESD mucosal defect spanning the angulus along the lesser curvature. ( **G** ) Partial closure (50–89% area reduction) was achieved. ( **H** ) SLE on post-ESD day 1 demonstrating stable wound condition without dehiscence, despite partial closure.

At the beginning of the procedure, several conventional clips were applied to the proximal and distal ends of the mucosal defect. This step narrowed the central width of the ulcer, effectively converting the defect from a circular shape to an oval one ( Fig. 2A,B ). Next, one or two MANTIS clips were applied at the center of the narrowed defect to bridge the widest gap ( Fig. 2C ). One MANTIS clip was applied when a single clip could adequately bridge the widest central gap; two clips were used when the remaining gap was too wide to be covered by a single clip, at the operator’s discretion. We aimed to grasp not only the mucosa but also the opposing muscular layers deeply. This technique was employed to approximate the tissues securely and eliminate dead space between the mucosa and the muscle base, a mechanical rationale recently reported to be valuable for achieving strong wound closure in the stomach. 13 This step was intended to prevent the loss of closure tension caused by the sloughing of ischemic necrotic mucosa, thereby preventing subsequent wound dehiscence. Finally, any remaining gaps were closed using conventional clips with the goal of achieving complete mucosal defect closure ( Fig. 2D ). For conventional clips, either SureClip (Microtech, Nanjing, China) or EZ Clip (Olympus) was selected at the discretion of the endoscopist.

All procedures were recorded, and the closure time and number of clips used were measured by reviewing the recorded videos. Closure time was defined as the duration between the loading of the first clip for closure and the deployment of the final clip. In cases involving intraoperative perforation, the time required for perforation repair was excluded from the closure time as it represents a separate procedural step that cannot be attributed to mucosal defect closure. However, clips used to repair the perforation were included in the total clip count because these clips approximate the surrounding tissue and may contribute to the mechanical stability of the subsequent mucosal defect closure. Representative cases of the complete and partial closure procedures are demonstrated in Video 1 .

### Histopathologic Diagnosis

One of four expert pathologists at our institution evaluated the histopathologic findings of the resected specimens. The final diagnoses were determined according to the Japanese Classification of Gastric Carcinoma, 15th edition. 14 The depth of the submucosal invasion was classified as SM1 when the invasive front was <500 μm from the muscularis mucosae and as SM2 when the depth of invasion was ≥500 μm.

### Propensity Score Matching

Propensity score matching (PSM) was performed to adjust for differences in the baseline characteristics between the closure and nonclosure groups. Based on previously reported risk factors for post-ESD bleeding, the following variables were used for matching: use of anticoagulant agents (warfarin or direct oral anticoagulants), use of antiplatelet agents (aspirin and/or thienopyridines), and defect major diameter. Additionally, SLE timing (postoperative day 1 or 3) was included as a matching covariate, as it may influence the frequency of hemostatic interventions during SLE. Matching was performed using a 1:1 nearest neighbor method with a caliper width of 0.2 to minimize selection bias and achieve balance between the groups.

### Outcome Measures

**Fig. 3:**
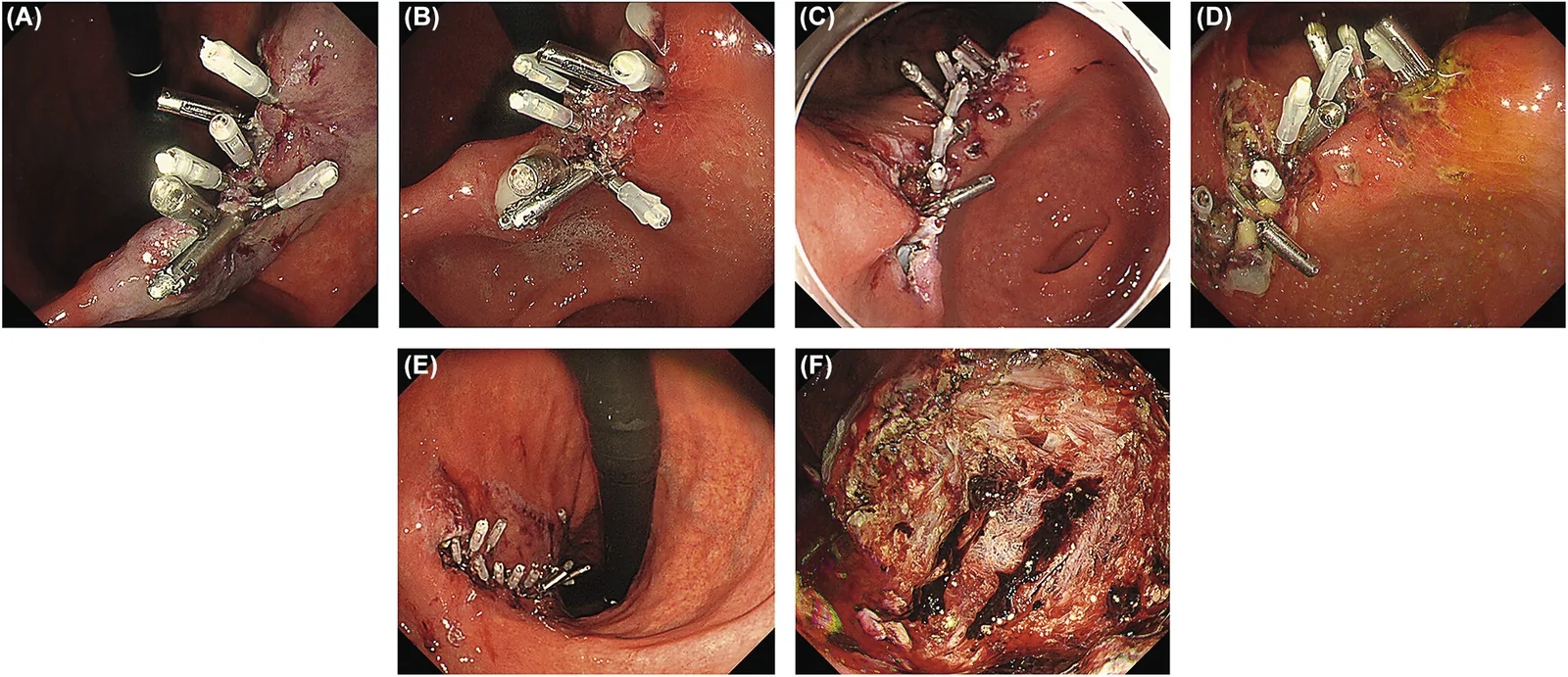
Closure stability categories at second-look endoscopy on post-endoscopic submucosal dissection day 1. ( **A** , **B** ) The defect was completely closed, and during the second-look endoscopy, nearly all clips remained in place, demonstrating sustained closure. ( **C** , **D** ) Partial dehiscence was observed, with some clips dislodged but the defect remaining mostly approximated. ( **E** , **F** ) Complete dehiscence with all clips dislodged was noted and the defect fully exposed.

As this was a pilot study, no sample size calculations were performed. The primary outcomes of this study were the technical success of the mucosal defect closure, closure time, and the stability of the closure at SLE. Technical success was defined as complete closure, namely (≥90% of the defect area closed) ( Fig. 2A–E ). For descriptive purposes, closure completeness was further classified as complete (≥90% closed, i.e., technical success), partial (50%–89% closed, i.e., Fig. 2F–H ), or failure (<50% closed). The stability of closure was assessed during SLE the day after ESD. To minimize subjective assessment bias, stability was strictly categorized based on the extent of wound maintenance compared to the findings immediately after the procedure: stable (sustained closure of ≥90% of the initially closed area with minimal or no clip detachment) ( Fig. 3A,B ); partial dehiscence (reopening of <50% of the initially closed area) ( Fig. 3C,D ); and complete dehiscence (reopening of ≥50% of the initially closed area or complete separation) ( Fig. 3E,F ).

Secondary outcomes, which were compared between the two groups in the PSM cohort, included the incidence of post-ESD bleeding, the frequency of hemostatic interventions during SLE, and adverse events. Post-ESD bleeding was defined as overt bleeding (hematemesis or melena) or a decrease in hemoglobin of more than 2 g/dL from the most recent level, confirmed by emergency endoscopy within 28 days after the ESD procedure. Hemostatic intervention at SLE was evaluated by reviewing medical records. SLE was routinely performed for all patients according to the protocol, regardless of symptoms. Hemostatic intervention during SLE was defined separately from post-ESD bleeding as the performance of prophylactic hemostasis (using clips or forceps) when adherent clots were observed on the ulcer bed or when oozing was triggered by mechanical irritation during the scheduled observation. In addition, adverse events, including post-coagulation syndrome and pneumonia, were assessed. Post-coagulation syndrome was defined as abdominal pain, fever, leukocytosis, or elevated C-reactive protein level. Pneumonia was defined as new-onset respiratory symptoms or pulmonary infiltrates during imaging within one day after ESD.

### Statistical Analysis

Continuous variables were presented as mean (standard deviation) or median [interquartile range], as appropriate based on the distribution of the data. Categorical variables were presented as numbers and percentages. Fisher’s exact test was used for nominal variables. For continuous variables with a normal distribution, a Student’s *t* test was applied for two-sample comparisons. When the data did not follow a normal distribution, the Mann–Whitney *U* test was used for two-sample comparisons. A *P* -value less than 0.05 was considered statistically significant for all tests. Statistical analyses were performed using EZR (version 1.60). 15

## Results

### Patient Characteristics

**Fig. 4:**
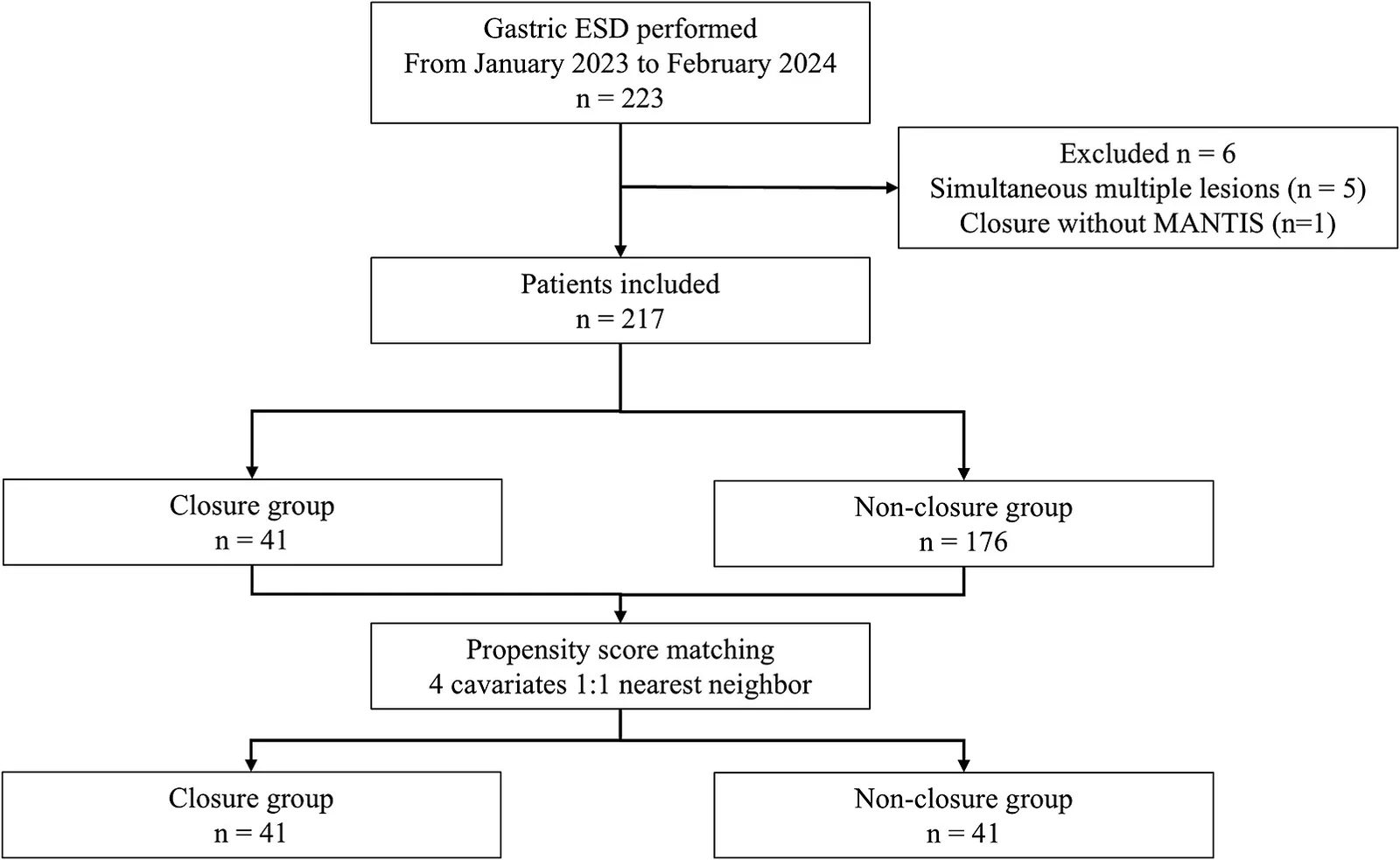
Patient flow diagram. Abbreviation; ESD, endoscopic submucosal dissection.

The patient flow is presented in Fig. 4 . A total of 217 patients were included in this study, comprising 41 in the closure group and 176 in the nonclosure group. The baseline characteristics of both groups are detailed in Table 1 . The proportion of patients taking anticoagulants or antiplatelet agents did not differ. Defect size was similar between the two groups. In the closure group, one case (2.4%) had a defect exceeding 60 mm (83 mm). In the nonclosure group, 9 cases (5.1%) had defects exceeding 60 mm (range 62–100 mm). Elevated-type morphology was more common in the closure group than in the nonclosure group ( *P* = 0.013). Expert endoscopists (>300 gastric ESDs) performed ESD in 14 patients (34.1%) in the closure group and 36 patients (20.5%) in the nonclosure group, with no statistically significant difference ( *P* = 0.067). Intraoperative perforation occurred in 1 patient (2.4%) in the closure group and 10 patients (5.7%) in the nonclosure group ( *P* = 0.69). These perforations were managed endoscopically with clips during the procedure. In the nonclosure group cases, clips were applied solely to repair the perforation, and the mucosal defect was left open without any attempt at closure. SLE was performed on postoperative day (POD) 1 in all closure group patients (100%) and in 151 nonclosure patients (85.8%). The remaining 25 nonclosure patients (14.2%) underwent SLE on POD 3 due to weekend scheduling, as ESD was performed on a Friday and SLE was performed on the following Monday. SLE timing differed significantly between groups ( *P* = 0.006). Delayed perforation was not observed in either group (0.0%).

**Table 1:** Baseline characteristics of all patients.

	Closure ( *n* = 41)	Nonclosure ( *n* = 176)	SMD	*P* value
Sex, *n* (%)			0.038	1.000
Male	33 (80.5)	139 (79.0)		
Female	8 (19.5)	37 (21.0)		
Age, y.o., median [IQR]	75 [71–80]	77 [70–82]	0.145	0.574
Anticoagulant drug, *n* (%)	2 (4.9)	20 (11.4)	0.239	0.265
Antiplatelet drug, *n* (%)	10 (24.4)	22 (12.5)	0.310	0.083
Lesion location, *n* (%)			0.161	0.736
Upper third	5 (12.2)	23 (13.1)		
Middle third	5 (12.2)	31 (17.6)		
Lower third	31 (75.6)	122 (69.3)		
Lesion circumference, *n* (%)			0.296	0.425
Anterior wall	8 (19.5)	28 (15.9)		
Posterior wall	4 (9.8)	32 (18.2)		
Lesser curvature	19 (46.3)	86 (48.9)		
Greater curvature	10 (24.4)	30 (17.0)		
Tumor size, mm, median [IQR]	12.00 [8.0–23.0]	12.00 [8.8–20.0]	0.035	0.993
UL, *n* (%)	2 (4.9)	8 (4.5)	0.016	1.000
Morphology, *n* (%)			0.390	0.013
Elevated	5 (12.2)	4 (2.3)		
Flat	36 (87.8)	172 (97.7)		
Invasion depth, *n* (%)			0.068	0.875
M	34 (82.9)	150 (85.2)		
SM1	4 (9.8)	14 (8.0)		
SM2	3 (7.3)	12 (6.8)		
Differentiation, *n* (%)			0.011	1.000
Differentiated	39 (95.1)	167 (94.9)		
Undifferentiated	2 (4.9)	9 (5.1)		
ESD operator, expert (>300 ESD), *n* (%)	14 (34.1)	36 (20.5)	0.311	0.067
Defect major diameter, mm, median [IQR]	32.0 [25.0–40.0]	32.0 [27.0–40.0]	<0.001	0.947
Defect minor diameter, mm, median [IQR]	26.0 [22.0–33.0]	25.0 [20.0–30.0]	0.180	0.298
Intraoperative perforation, *n* (%)	1 (2.4)	10 (5.7)	0.165	0.694
SLE timing, *n* (%)			0.575	0.006
POD 1	41 (100.0)	151 (85.8)		
POD 3	0 (0.0)	25 (14.2)		

Abbreviations: IQR, interquartile range; ESD, endoscopic submucosal dissection; UL, ulceration; SM, submucosa; SMD, standardized mean difference; SLE, second-look endoscopy; POD, postoperative day. *P* values: Fisher’s exact test for categorical variables; Mann -Whitney *U* test for continuous variables.

### Technical Success and Stability

Table 2 shows the procedural details of the closure group. The median closure time was 600 [450–882] seconds. Two MANTIS clips were used in 4 cases (9.8%). These cases tended to have larger defect major diameters (45.5 mm [IQR 37.3–53.5] vs. 32.0 mm [IQR 25.0–39.0]; *P* = 0.055) and had significantly longer closure times (1155 sec [IQR 1020–1186] vs. 575 sec [IQR 450–762]; *P* = 0.015) compared with single-clip cases. Technical success (complete closure, ≥90% of the defect area closed) was achieved in 92.7% (38/41) of the cases. Partial closure (50%–89%) occurred in 7.3% (3/41). The three cases of partial closure were located at the angulus (defect size: 50 mm), the cardia (defect size: 24 mm), and the anterior wall of the antrum (defect size: 53 mm). Closure failure (<50% closed) did not occur. Regarding the stability of the closure defect at SLE on post-ESD day 1, sustained closure was maintained in 87.8% (36/41) of the cases. Partial dehiscence occurred in 3 cases (7.3%). These cases were located at the angulus (lesser curvature, defect size: 41 mm), the pyloric ring (lesser curvature, defect size: 33 mm), and the antrum (lesser curvature, defect size: 25 mm). Complete dehiscence occurred in 2 cases (4.9%). These involved one case at the antrum (greater curvature, defect size: 32 mm) and one large defect at the lower body (anterior wall, defect size: 83 mm).

**Table 2:** Procedural details of the closure group.

	Closure ( *n* = 41)
MANTIS clip, *n* , median [IQR]	1 [1–1]
1 clip, *n* (%)	37 (90.2)
2 clips, *n* (%)	4 (9.8)
SureClip, *n* , median [IQR]	2 [2–3]
EZ Clip, *n* , median [IQR]	4 [3–5]
Closure time, s, median [IQR]	600 [450–882]
Technical success (complete closure, ≥90%), n (%)	38 (92.7)
Partial closure (50–89%), *n* (%)	3 (7.3)
Closure failure (<50%), *n* (%)	0 (0.0)
Closure stability at SLE, *n* (%)	
Sustained (≥90% maintained)	36 (87.8)
Partial dehiscence (<50% reopening)	3 (7.3)
Complete dehiscence (≥50% reopening)	2 (4.9)

Abbreviations: IQR, interquartile range; SLE, second-look endoscopy. Closure time: duration from first clip loading to final clip deployment; perforation repair time excluded.

### Propensity Score Matching Procedure

After PSM, 41 patients were included in each group. As shown in Table 3 , *P* values exceeded 0.05 for all baseline characteristics. Standardized mean differences (SMDs) remained below 0.1 for 8 of the 16 baseline variables, including all four covariates entered into the matching model (anticoagulant use, antiplatelet use, defect major diameter, and SLE timing; SMD ≤ 0.015 for each), whereas SMDs exceeded 0.1 for eight nonmatched variables: age (0.338, the maximum value), morphology (0.264), intraoperative perforation (0.13), defect minor diameter (0.124), lesion location (0.110), operator experience (0.105), ulceration (0.102), and invasion depth (0.102). The c-statistic of the propensity score model was 0.642 (95% CI 0.550–0.733). The median number of clips used per closure procedure was 1 [1–1] for the MANTIS clip, 2 [2–3] for SureClip, and 4 [3–5] for EZ Clip.

**Table 3:** Baseline characteristics after propensity score matching.

	Closure ( *n* = 41)	Nonclosure ( *n* = 41)	SMD	*P* value
Sex, *n* (%)			0.063	1
Male	33 (80.5)	34 (82.9)		
Female	8 (19.5)	7 (17.1)		
Age, y.o., median [IQR]	75 [71–80]	77 [73–82]	0.338	0.309
Anticoagulant drug, *n* (%)	2 (4.9)	2 (4.9)	<0.001	1.000
Antiplatelet drug, *n* (%)	10 (24.4)	10 (24.4)	<0.001	1.000
Lesion location, *n* (%)			0.110	0.881
Upper third	5 (12.2)	6 (14.6)		
Middle third	5 (12.2)	6 (14.6)		
Lower third	31 (75.6)	29 (70.7)		
Lesion circumference, *n* (%)			0.093	1
Anterior wall	8 (19.5)	7 (17.1)		
Posterior wall	4 (9.8)	5 (12.2)		
Lesser curvature	19 (46.3)	19 (46.3)		
Greater curvature	10 (24.4)	10 (24.4)		
Tumor size, mm, median [IQR]	12.0 [8.0–23.0]	12.0 [10.0–21.0]	0.055	0.593
UL, *n* (%)	2 (4.9)	3 (7.3)	0.102	1
Morphology, *n* (%)			0.264	0.432
Elevated	5 (12.2)	2 (4.9)		
Flat	36 (87.8)	39 (95.1)		
Invasion depth, *n* (%)			0.102	1
M	34 (82.9)	35 (85.4)		
SM1	4 (9.8)	4 (9.8)		
SM2	3 (7.3)	2 (4.9)		
Differentiation, *n* (%)			<0.001	1
Differentiated	39 (95.1)	39 (95.1)		
Undifferentiated	2 (4.9)	2 (4.9)		
ESD operator, expert (>300 ESD), *n* (%)	14 (34.1)	12 (29.3)	0.105	0.813
Defect major diameter, mm, median [IQR]	32.0 [25.0–40.0]	32.0 [26.0–40.0]	0.015	0.915
Defect minor diameter, mm, median [IQR]	26.0 [22.0–33.0]	25.0 [20.0–30.0]	0.124	0.377
Intraoperative perforation, *n* (%)	1 (2.4)	2 (4.9)	0.13	1
SLE timing, POD 1, *n* (%)	41 (100.0)	41 (100.0)	<0.001	1

The c-statistic of the propensity score model was 0.642 (95% CI 0.550–0.733).

Abbreviations: IQR, interquartile range; ESD, endoscopic submucosal dissection; UL, ulceration; SM, submucosa; SMD, standardized mean difference; SLE, second-look endoscopy; POD, postoperative day. *P* values: Fisher’s exact test for categorical variables; Mann -Whitney *U* test for continuous variables.

### Post-ESD Hemostasis and Adverse Events

Table 4 shows the clinical outcomes after PSM. The frequency of additional hemostatic interventions during SLE was lower in the closure group than in the nonclosure group (24.4% [10/41] vs. 58.5% [24/41], *P* = 0.003). Post-ESD bleeding occurred in 0 patients (0.0%) in the closure group and 2 patients (4.9%) in the nonclosure group ( *P* = 0.494). Postcoagulation syndrome occurred in 7.3% (3/41) of the patients in the closure group and 4.9% (2/41) in the nonclosure group ( *P* = 1.00). Pneumonia occurred in one patient in each group (2.4%, *P* = 1.00).

**Table 4:** Clinical outcomes after propensity score matching.

	Closure ( *n* = 41)	Nonclosure ( *n* = 41)	SMD	*P* value
Hemostatic intervention at SLE, *n* (%)	10 (24.4)	24 (58.5)	0.739	0.003
Delayed bleeding, *n* (%)	0 (0.0)	2 (4.9)	0.32	0.494
Post-coagulation syndrome, *n* (%)	3 (7.3)	2 (4.9)	0.102	1.000
Pneumonia, *n* (%)	1 (2.4)	1 (2.4)	<0.001	1.000

Abbreviations: SLE, second-look endoscopy; SMD, standardized mean difference. *P* values: Fisher’s exact test.

## Discussion

This pilot study demonstrated that mucosal defect closure using the MANTIS clip is technically feasible and stable. Technical success (complete closure) was achieved in 92.7% of the cases, with no closure failure (<50% area reduction) observed in any patient, and closure was sustained or partially maintained in 95.1% of the cases during SLE. We attribute this stability to two factors: the device’s gripping power with its bifurcated jaw design and our specific closure technique of grasping the muscular layer deeply to eliminate dead space between the mucosa and the muscle. A recent case report suggests that crimping the muscular layer to minimize dead space supports strong gastric closure. 13 This mechanism may apply directly to our three partial closure cases involving large defects (≥50 mm) or the cardia. While conventional clips typically fail under such high anatomical tension, the MANTIS clip’s superior grasping power and rotational control enabled at least a partial closure to safely stabilize the wounds. Furthermore, unlike complex endoscopic suturing systems, the MANTIS clip operates similarly to conventional clips. In this study, the closure procedure added about 10 min to the total operation time. This was faster than the reopenable-clip over line method (ROLM) (reported as median 30 min), 8 and endoscopic hand suturing (EHS) (reported median 36 min). 5 This shorter closure time suggests that the MANTIS clip may offer a procedural advantage over more complex closure systems.

The frequency of hemostatic interventions during SLE was chosen as a secondary outcome because it serves as a practical surrogate for ulcer bed stability, providing a direct, procedure-level indicator of whether mucosal closure effectively shields the wound from mechanical stimulation. Regarding the delayed bleeding, no difference was observed between the closure group and the nonclosure group. This was likely due to the study being underpowered to detect differences in low-frequency events within a predominantly low-risk cohort. However, the stable closure has a clinical benefit: the need for hemostatic intervention during SLE was reduced from 58.5% to 24.4%. This finding suggests that MANTIS clip closure provides a physical shield, protecting the ulcer bed from mechanical stimulation during the SLE procedure. Given that SLE interventions do not consistently prevent delayed bleeding and may serve as a mechanical trigger for iatrogenic hemorrhage, minimizing such interventions is relevant. These results provide a rationale to investigate whether stable closure could support the omission of SLE, particularly in high-risk populations.

The cost-effectiveness of the closure strategy was assessed using the incremental cost-effectiveness ratio (ICER), calculated as the difference in cost between the two strategies divided by the difference in the delayed bleeding rate (ICER = Δcost/Δincidence). 16 17 In Japan, clipping devices are not reimbursed under the national fee schedule and represent a direct institutional expense; based on the median device usage in this cohort (one MANTIS clip at 113.3 USD/106.3 EUR [17,000 JPY], two SureClips at 23.3 USD/21.9 EUR [3500 JPY] each, and four EZ Clips at 4.7 USD/4.4 EUR [700 JPY] each), the incremental device cost per closure case was 179 USD/168 EUR (26,800 JPY). Applying the PSM-derived difference in delayed bleeding rates (0% vs. 4.9%), the ICER was approximately 3,663 USD/3,434 EUR (549,400 JPY) per bleeding event prevented. Monetary conversions are based on representative exchange rates of 1 USD = 150 JPY and 1 EUR = 160 JPY, reflecting average levels during the study period. This finding suggests that universal closure is not cost-effective in an unselected, predominantly low-risk population. However, in high-risk patients (e.g., those on anticoagulant therapy) who have higher baseline bleeding rates, the economic profile of selective MANTIS clip closure would become more favorable.

This study has several limitations. First, the retrospective, single-center design is susceptible to selection bias that PSM cannot fully overcome. Second, as noted, the study is underpowered, and therefore, the secondary endpoint of delayed bleeding in this low-risk cohort could not be determined. Third, there was potential operator bias. To ensure technical consistency, all closure procedures were performed by expert endoscopists, whereas the nonclosure group included procedures performed by various endoscopists, including trainees. Therefore, the outcomes in the closure group might be partially attributable to the operator’s technical skill in ESD, rather than the closure device itself. Fourth, the data for hemostatic interventions during SLE were from a retrospective chart review, and the threshold for intervention was not standardized but left to the individual endoscopist’s discretion. Fifth, the limited size of the closure group necessitated restricting the propensity score model to four covariates, which may have resulted in incomplete adjustment for potential confounders not entered into the model. Consistent with this, absolute SMDs after matching exceeded 0.1 for age, morphology, intraoperative perforation, defect minor diameter, lesion location, operator experience, ulceration, and invasion depth, indicating residual imbalance in these characteristics. Sixth, because our study involved an unselected population, the closure group likely included relatively straightforward mucosal defects that could have been adequately managed with conventional clips alone. This mix of cases may have diluted the specific clinical benefits and added value of the MANTIS clip, which are likely more pronounced in higher-risk or more challenging lesions. Seventh, the clinical significance of partial closure remains uncertain. Although all three partial closure cases remained stable without dehiscence at SLE, whether a 50%–89% reduction in the defect area confers a protective effect against post-ESD adverse events comparable to complete closure has not been established; for this reason, we did not classify partial closure as a technical success. Evidence from other settings is, if anything, conflicting with such an assumption: in colorectal ESD, complete closure—but not partial or no closure—was associated with a lower delayed bleeding rate in patients receiving anticoagulants. 18 Eighth, the direct association between hemostatic interventions during SLE and other clinical endpoints, such as hospital stay or re-intervention, was not evaluated. Finally, this study did not evaluate long-term outcomes, such as delayed ulcer healing or stricture formation.

## Conclusion

This pilot study demonstrated that MANTIS clip-assisted closure is technically feasible and stable. Although it did not reduce delayed bleeding in this low-risk cohort, stable closure revealed a secondary benefit: a reduction in hemostatic interventions during SLE. This finding suggests that stable closure may simplify postoperative management by supporting the omission of SLE. Future prospective studies are warranted to evaluate a management strategy that combines stable MANTIS clip closure with the omission of routine SLE in high-risk populations.
